# Efficiency of multiple imputation to test for association in the presence of missing data

**DOI:** 10.1186/1753-6561-1-s1-s24

**Published:** 2007-12-18

**Authors:** Pascal Croiseau, Claire Bardel, Emmanuelle Génin

**Affiliations:** 1 Universite Paris-Sud UMR-S535, Villejuif, 94817 France; 2 INSERM #U535, BP 1000, Villejuif, 94817 France; 3 UMR 5145 – Génétique des Populations Humaines – CNRS MNH, Université Paris VII, 17 Place du Trocadero, Paris, 75016 France

## Abstract

The presence of missing data in association studies is an important problem, particularly with high-density single-nucleotide polymorphism (SNP) maps, because the probability that at least one genotype is missing dramatically increases with the number of markers. A possible strategy is to simply ignore the missing data and only use the complete observations, and, consequently, to accept a significant decrease of the sample size. Using Genetic Analysis Workshop 15 simulated data on which we removed some genotypes to generate different levels of missing data, we show that this strategy might lead to an important loss in power to detect association, but may also result in false conclusions regarding the most likely susceptibility site if another marker is in linkage disequilibrium with the disease susceptibility site. We propose a multiple imputation approach to deal with missing data on case-parent trios and evaluated the performance of this approach on the same simulated data. We found that our multiple imputation approach has high power to detect association with the susceptibility site even with a large amount of missing data, and can identify the susceptibility sites among a set of sites in linkage disequilibrium.

## Background

Association studies are often faced with a problem of missing data, either in the form of a missing genotype or in the form of unknown phase. There is a temptation to simply ignore the missing data and only use the complete and phase-known observations, but it has been shown that this can induce bias and/or loss in power [1,2]. When the level of missing data differs from one marker to another, focusing only on the complete data in the analysis will make it very difficult to compare different markers, and may lead to false conclusions regarding which marker(s) are most likely to explain the detected association and the location of sites involved in disease susceptibility. Indeed, if the disease susceptibility site is among the studied sites but is poorly genotyped, it is possible that a marker in linkage disequilibrium with this site will obtain a better association score than the disease susceptibility site itself.

Multiple imputation (MI) might provide an interesting and convenient solution to the problem. The idea of the method is to fill in missing data by values that are predicted by the observed data. The observed data set containing missing values is replaced by a small number of simulated complete data sets (e.g., 3–10) that are analyzed by standard methods, and the results are combined to produce estimates and confidence intervals that incorporate the missing-data uncertainty [3]. We recently proposed a MI approach to deal with missing phase and missing genotype in the context of family-based association studies [4]. In this paper, we evaluate the performance of the MI approach in detecting disease susceptibility sites using the Genetic Analysis Workshop 15 (GAW15) simulated data, where we removed some genotypes to generate different levels of missing data.

## Methods

The first 500 families of each of the 100 replicates simulated for GAW 15 (Problem 3) were considered and case-parent trios were obtained by selecting both parents and the first affected sib in each sibship. Using the answers, we chose to focus on chromosome 6 in the region containing both the DR and C loci, and we were interested in detecting the effect of the C locus. In this region, nine SNPs (including locus C) were selected. A tenth biallelic locus, corresponding to the DR locus in which the lower risk alleles DR1 and DRX were pooled, was added.

Starting from the complete data, we randomly deleted genotypes at locus C to generate different levels of missing data, but we kept the complete information at the other loci. To limit the impact of variation in the patterns of missing data between replicates, we chose to delete the genotypes of the same individuals in different replicates and to have the same proportion of missing data for different family members. The proportion of missing data was varied between 5 and 50 percent. A MI algorithm [3] that we recently developed to deal with case-parent trio data [4] was performed for each sample.

Briefly, the principle of this method is to fill in missing data with values that are predicted by the observed data. For each family containing a missing value, a haplotype is selected among all the compatible haplotypes with a probability given by the current posterior distribution (at the starting point, this posterior distribution comes from an expectation maximization algorithm). Population haplotype frequencies are then updated using the new posterior distribution that comes from the current complete data file. These two steps are iterated a large number of times and when the stationary distribution is reached (here after a burning period of 1000 iterations) a small number of complete data sets (here this number was nine) are selected every 1000 iterations. Each simulated complete data set is analyzed separately and the results are combined to produce estimates that incorporate missing data uncertainty [3,5,6].

Inference of missing values is performed using observed genotypes, affection status data, and family structure.

In the present study, analysis was performed using a conditional logistic regression method [2,7,8] that compares the genotype of an affected child (case, *c*) to the three possible genotypes that can be formed by the untransmitted parental alleles (pseudo controls, *pc*_*j *_with *j *= 1 to 3). The likelihood of the data is written as a linear function:

$$L_{1}=\prod_{k}\frac{\exp(\beta_{0}+\beta_{1}x_{1k_{c}}+...+\beta_{n}x_{nk_{c}})}{\exp(\beta_{0}+\beta_{1}x_{1k_{c}}+...+\beta_{n}x_{nk_{c}})+\sum_{j=1}^{3}\exp(\beta_{0}+\beta_{1}x_{1k_{pc_{j}}}+...+\beta_{n}x_{nk_{pc_{j}}})},$$

where $x_{ij_{k}}$ is an indicator taking value 1 if case or pseudo control *j *in family *k *has genotype *i*, and 0 otherwise. *β*_i _= log OR_i_, with *β*_0 _being the baseline risk for reference genotype. Under the null hypothesis of no association, the log likelihood is simply: Ln(L_0_) = *β*_0_.

For each of the *m *complete data files *i*, we calculate the likelihood ratio test *d*_*i *_as

*d*_*i *_= 2[ln(*L*_1_) - ln(*L*_0_)]

and combine the *d*_*i *_across data sets using the method described in Schafer [5] and Rubin and Little [6]. The power to detect the association with each locus was obtained by computing the proportion of replicates for which the test is significant at a nominal level of 5% at each marker. Given the fact that the DR locus is located in the studied region and has a strong effect on the disease, we also performed tests conditional on the DR locus to see if the association remains at the other loci after accounting for the DR locus effect.

## Results

On the complete data, six out of the ten markers were associated with the disease in most of the replicates (see Table 1). When conditioning on the DR locus, many of these associations are no longer detected except for locus C, and to a lesser extent, SNP4.

**Table 1:** Proportion of replicates in which each marker gives a significant association test

Locus	Association test	Association test conditional on DR
1	0.19	0.05
2	0.6	0.03
3	1	0.18
4	1	0.33
C	1	0.83
6	1	0.2
7	0.97	0.09
8	0.09	0.06
9	0.16	0.06
DR	1	X

**Figure 1:**
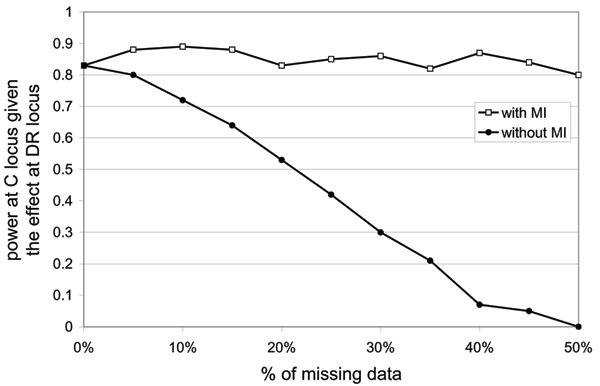
**Power to detect the effect of locus C in diseasesusceptibility**. Comparison of the power to detect the C locus effect with and without MI in function of the percentage of missing data at locus C. Power of the test accounting for the DR locus effect is computed over the 100 replicates using the first 500 families.

**Figure 2:**
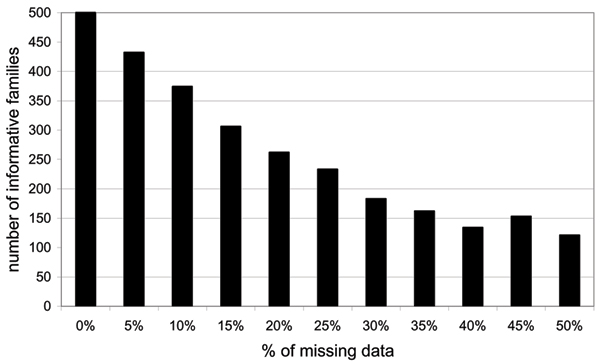
Number of informative families at locus C in function of the percentage of missing data.

As expected, an increase in the percentage of missing data at locus C leads to a decrease in the power to detect the effect of the C locus when MI is not used (Figure 1). Indeed, an important reduction in the sample size is observed as the proportion of missing data increases, from 500 families in the absence of missing data to less than 150 families when there is 50% of data missing at locus C (Figure 2). Interestingly, when using the MI approach, no power loss is observed (see Figure 1 with MI), and even with 50% of data missing, the power remains above 80%.

**Figure 3:**
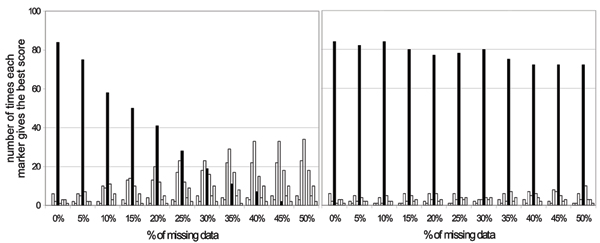
**Number of times each marker gives the best scorefor the association test**. Association test were performed given the effect of DR for different percentage of missing data at locus C. For each percentage of missing data, the number of replicates among the 100 replicates in which each of the ten markers gives the best association score is reported. Each bar represents a different marker and the black bar represents SNP C. Left, Results obtained when trios with missing data are discarded. Right, Results obtained using the MI approach.

Once association is detected, one is often interested in identifying the site (or sites) that are most likely involved in disease susceptibility. This could be done by identifying the site that exhibits the most significant association. Figure 3 shows the number of times each of the ten markers gives the best score for the association test conditional on DR. In the absence of missing genotype data, locus C gives the highest score in almost all the replicates, as expected. However, when the percentage of missing data at locus C increases, and missing data are not taken into account (see Figure 3a), other loci more frequently exhibit the highest significance, particularly SNP4, which is in strong LD with C (D' = 0.84, *r*^2 ^= 0.65). For 50% missing data, locus C is not even identified in a single replicate, whereas SNP4 is identified in 34 out of the 100 replicates. This latter locus is identified as the most significant one more often than locus C for levels of missing data above 30%. However, using the MI approach (see Figure 3b), locus C is identified as the most significance locus in over 70% of the replicates, even with a strong percentage of missing data. With 50% missing data, locus C is the most significant locus in 72 out of the 100 replicates.

## Discussion

In this paper, we used the GAW15 data to show the impact of missing data on both the power to detect an association and the prediction of the disease susceptibility site. By contrasting findings with and without missing data, we were able to gain some insights regarding the performance of our MI approach. As expected, not accounting for missing data can lead to a significant loss in power, and errors in the prediction of the disease susceptibility location. We have demonstrated that MI is an interesting and efficient approach to limit power losses and prediction errors. Indeed, using this approach, we observed only very limited losses in power for missing data levels of up to 50%. In terms of localization of the disease susceptibility site, the performance of the method is also very accurate, because the true disease susceptibility site is identified in the majority of replicates when using MI.

The effect of missing data on power and localization of the disease susceptibility locus is small for levels of missing data below 10%, but above 10% it can be a real problem. With the current genotyping technologies, genotype failures are considerably less than 10%. However, when using family data, availability of all members of the family for genotyping, particular parents, is not always guaranteed, and higher rates of missing data might then be encountered. In these situations, MI might be a useful way to get maximum benefit of the sample.

In the present study, we chose to simulate missing data only at the disease susceptibility site. Although this might not be very realistic, because missing data will generally be found for different markers, our results demonstrate that even under this scenario in which the individual signal at the true disease locus is smaller than at a fully genotyped marker in strong linkage disequilibrium, MI performs extremely well at identifying the true disease susceptibility locus.

Several alternative methods have been developed to infer missing data from the rest of the data. In the context of family-based association studies, specific methods have been developed mostly based on likelihood approaches. One problem with these methods, and their corresponding software, is their lack of flexibility. Different applications of these methods are required if, for example, one also wants to account for environmental risk factors and potential gene × environment interactions in the analysis. In this context, it is of interest to develop methods such as MI that work in the framework of traditional statistical packages and allow the inclusion of arbitrary genetic and/or environmental predictor variables in a model. Indeed, the MI approach generates complete data sets that can be individually analyzed using, for instance, conditional logistic regression with any available covariates. Results then need to be combined using the methods described in Little and Rubin [6].

## Conclusion

In conclusion, multiple imputation appears to be an efficient method to deal with missing data. It limits power reduction to detect association. Interestingly, it also performs well in identifying the most likely locus involved in disease susceptibility among several sites in linkage disequilibrium, even if missing data is concentrated on this site.

## Competing interests

The author(s) declare that they have no competing interests.
